# Complete Genome Sequence of *Sedimenticola thiotaurini* Strain SIP-G1, a Polyphosphate- and Polyhydroxyalkanoate-Accumulating Sulfur-Oxidizing Gammaproteobacterium Isolated from Salt Marsh Sediments

**DOI:** 10.1128/genomeA.00671-15

**Published:** 2015-06-18

**Authors:** Beverly E. Flood, Daniel S. Jones, Jake V. Bailey

**Affiliations:** University of Minnesota, Department of Earth Sciences, Minneapolis, Minnesota, USA

## Abstract

We report the closed genome sequence of *Sedimenticola thiotaurini* strain SIP-G1 and an unnamed plasmid obtained through PacBio sequencing with 100% consensus concordance. The genome contained several distinctive features not found in other published *Sedimenticola* genomes, including a complete nitrogen fixation pathway, a complete ethanolamine degradation pathway, and an alkane-1-monooxygenase.

## GENOME ANNOUNCEMENT

The genus *Sedimenticola* is the sole described subclade within a large clade of unclassified bacteria that includes a number of sulfur-oxidizing endosymbionts of marine invertebrates endemic to sulfidic biomes (1). However, the type strain *Sedimenticola selenatireducans* AK4OH1 was initially described as a strict anaerobe capable of coupling aromatic degradation with anaerobic respiration to include respiring selenate to selenite (2). Recently, several studies determined that the *Sedimenticola* spp. are indeed sulfur-oxidizing bacteria as their phylogeny infers (3–5). *Sedimenticola thiotaurini* strain SIP-G1 (ATCC = BAA − 2640^T^; DSMZ = 28581^T^) was isolated from sediments of the Sippiwissett Salt Marsh, Falmouth, MA, USA. This strain had the capacity to couple the oxidation of reduced sulfur compounds with autotrophic growth under hypoxic and anaerobic conditions. Anaerobic electron acceptors include NO_3_^−^, NO_2_^−^, ClO_4_^−^, ClO_3_^−^, trimethylamine *N*-oxide, dimethyl sulfoxide, SeO_4_^−^, and BrO_3_^−^. When in the presence of excess organics, especially acetate, this strain produces polyphosphate granules prior to accumulating large quantities of polyhydroxyalkanoates.

More than 12 µg of genomic DNA from a batch culture grown aerobically on modified marine broth 2216 was extracted and purified via Qiagen’s Genomic-tip 500/G according to the manufacturer’s instructions. Size selection with a 3-kb cutoff was performed to obtain ~20-kb insert target continuous long reads using BluePippin technology (Sage Science). The reads were then sequenced via 120-min movies on eight SRMT cells using P4-C2 chemistry on a PacBio RS II sequencer (Mayo Clinic Bioinformatics Core, Rochester, MN, USA).

The genome filtering, assembly, and reassembly were performed using tools within SMRT Analysis v2.1 (6). Raw reads (~13 Gbp total) were filtered to remove SMRT bell adapters and short (<100-bp) and low-quality (80% accuracy) reads. *De novo* assembly using HGAP version 3 was performed with self-corrected long reads with a minimum length cutoff of 10,000 bp resulting in ~90× coverage of the bacterial genome and ~30× coverage of a plasmid genome. The genomes were circularized using Minimus2 and polished with Quiver in two consecutive rounds to remove any remaining indels (6). The final circularized genome of *S. thiotaurini* SIP-G1 was 3,928,944 bases with a G+C content of 57.64%. The average coverage was 294× with 100% consensus concordance. The final circularized genome of the plasmid was 33,340 bases with an average G+C content of 54.31%. The average coverage was 143× with 100% consensus concordance. Annotation was performed using the NCBI Prokaryotic Genomes Annotation Pipeline as well as the JGI Integrated Microbial Genomes Pipeline (7).

Distinguishing genomic features not found in the sister taxa *Sedimenticola selenatireducens* AK4OH1, *Sedimenticola selenatireducens* CUZ, and *Sedimenticola* NSS, also known as “*Dechloromarinus chlorophilus* NSS,” include the presence of a complete nitrogen fixation pathway, a complete ethanolamine degradation pathway, urease, and an alkane-1-monooxygenase. *S. thiotaurini* SIP-G1 lacks complete aerobic and anaerobic aromatic degradation pathways recently investigated in the *Sedimentico*la (8), methylamine dehydrogenase, and ribulose-1,5-bisphosphate form I. Instead, *S. thiotaurini* SIP-G1 has a complete Calvin-Benson-Bassham cycle with ribulose-1,5-bisphosphate form II to catalyze CO_2_ fixation. The plasmid in *S. thiotaurini* SIP-G1 carries few, if any, functional genes outside of plasmid maintenance genes.

### Nucleotide sequence accession numbers.

The assembly and annotation files were deposited in GenBank under the accession numbers CP011412 and CP011413. The annotated genome is also available through the Integrated Microbial Genomes database, taxon ID 2609459601.
